# Medical News and Miscellany

**Published:** 1891-05

**Authors:** 


### Medical News and Miscellany.

Dr. L. L. Shropshire, of San Antonio, is attending the New York Polyclinic, and instructs us to send the JOURNAL there for several months.

Dr. S. E. Carrington, of Franklin, Texas, is at the New Orleans Polyclinic,—requests the JOURNAL forwarded there; "can't do without it."

Dr. B. F. Gibson, of Midway, was married recently to Miss Irma Mitchell, and has been appointed physician to the Huntsville Penitentiary.

Antifebrin in Soft Chancre.—Dr. Bazilevich recommends antifebrin as an excellent remedy for chancroids, to be dusted on twice daily.—*Ex.*

Will Sell my Place and Practice.—Growing railroad town of 2000 inhabitants; two physicians; large country practice. I want to quit at once, but will stay till January next, to collect and assist successor in starting. Approximate value, $1500. Address this office.

Death of Dr. Graham.—The JOURNAL is pained to announce the death of Dr. L. J. Graham, of Henderson, a distinguished member of the profession and a model gentlemen. He died at his home, April 15, 1891, after an illness of two weeks, the result of la grippe. He was a graduate of Jefferson Medical College, class of 1855.

The Positive Pole in Menorrhagia and Metrorrhagia.—Dr. A. G. Goelet, in *N. Y. Medical Record* of March 28, calls attention to a number of cases cured by the use of a strong galvanic current. A suitable electrode is introduced to the fundus, and allowed to remain about five minutes. This operation is repeated every two or three days.

**Dr. G. W. Kerr**, of Waelder, ex-president of the West Texas District Medical Association, died in April, of pneumonia. He was one of the purest and best men in the State Association, and his death is a great loss to the profession.

---

**Dr. W. A. Rape**, of Ballinger, had the misfortune to lose his office by fire on the night of the 7th of April ult., including all his furniture, fixtures, books, instruments, etc., and even his diploma. Fortunately the diploma had been registered in the office of the county clerk, and also in Nacogdoches county. We sympathize with the doctor in his loss.

---

**The Dangerous Properties of Dr. Koch's Lymph** will be removed in the future, thanks to the researches of one of the Professor's assistants. Dr. Weyl has discovered a means of detecting the poisonous elements pointed out by Prof. Virchow, and can eliminate them by a process of his own invention.—*London Correspondent American Practitioner and News, March 14, '91.*

---

**Notice.**—The archives of the Texas State Medical Association its records, books and seal, and the large lot of Transactions, have been turned over to Dr. H. A. West, the newly elected Secretary, at Galveston, and to him all communications should be addressed, as to matters relating to the Association. Remittances for dues must be made to Dr. J. Larendon, Treasurer, Houston, Texas.

---

**Turning the Joke.**—The *Waco Day* says that during the session of the State Medical Association at city hall in that city, a member thinking to "poke fun" at an old darkey, asked him if he could tell him where the *Lime Kiln Club* was in session; and that the old darkey said, he "didn't know 'zactly," but he "*b'leeved* it was at the city hall."

Had the old darkey gotten wind of anything going on in the Medical Association that suggested *lime?*

---

**A National Medical Temperance Society.**—Dr. W. A. Morris' paper before the Texas State Medical Association, on the Abuses of Alcohol and the Responsibility of the Profession, was as timely as it was able. The profession is waking up to a realizing sense, not only of its responsibility for much intemperance, but to their duty to take a leading part in reform. Dr. N. S. Davis—all honor to him—called a meeting of physicians, while in Washington, interested in the subject, for the purpose of establishing a National Medical Temperance Society. We shall await developments with much interest.

---

**Removals.**--D. J. H. Evans has removed from Alto, Cherokee county, to Palestine.

Dr. H. B. Butcher, from Laredo to Montmoreles, Nueva Leon, Mexico.

Dr. A. M. Clinkscales, from Hubbard City, Texas, to Vanita, Ind. Ter.

Dr. W. J. Miller, of Massey, after returning from New York, where he attended a course at Bellevue and the Polyclinic, removed to Itaska to practice.

Dr. C. M. Scogin has removed from Throckmorton to Albany, Texas, and has formed a copartnership with Dr. W. M. Powell.

---

**Dr. Will B. Davis**, late of Grapevine, Tarrant county, and who will be remembered by all old members of the Association, especially in connection with his brilliant success in hemorrhoids with carbolic acid by injection, and the sensation his report made at the Tyler meeting in 1883,—removed some time ago to Puebla, Colorado. The good people there were not long in recognizing his true worth, and his claims to confidence, for he has worked into a big paying practice, and has been made city physician. The papers give a very complimentary notice of him and publish a good cut of him. We extend to him our hearty congratulation.

---

**Death of Dr. Paine.**—Dr. F. T. Paine, of Comanche, Tex., died April 16, 1891. Dr. Paine was well known in connection with his researches in electro-therapeutics; was one of the pioneers in Texas in that branch of study, and contributed some valuable papers to the literature of the subject. He died at an advanced age, after a long life of usefulness and labor in the cause of humanity. He was an old member of the Texas State

Medical Association, and will be much missed at the annual meetings. Dr. C. F. Paine, of Comanche, a prominent physician, is his son. We knew Dr. Paine well and loved him; he was a true friend, a noble man and a good and conscientious practitioner of the healing art.

---

**Texas Medical College.**—The Texas Medical College will soon be replaced by the Medical Branch of the University of Texas. An appropriation was made by the last legislature to finish, furnish and equip the magnificient building now nearly completed. The Faculty will be elected at an early day. The institution will be first-class in every respect. The fees will be moderate. The advantages will be such that no Texas medical student can afford to leave the State. The announcement will be issued as soon as practicable, and the session begin about 1st October next.

H. A. West, M. D.,
Dean Texas Medical College.

---

**New Journals.**—The *Journal of Gynecology*, a monthly devoted to gynecology, obstetrics and abdominal surgery, Vol. I., No. 1, edited by Charles N. Smith, M. D., Toledo, Ohio. Subscription, $1.50 per annum; single copies, 20 cents.

The first issue is very creditable in appearance and character of contents. It starts out with the promise of giving its readers from forty-eight to sixty-four pages of pure reading matter, with no reading notices or insert advertisements. The leading features of the *Journal of Gynecology* will be the original communications, and bibleographical index. It has a long list of prominent contributors, three of whom are from Texas: W. T. Baird, Hadra and Ghent. The only objection to the new periodical is its cheapness. One dollar and fifty cents per annum will not sustain it, morally nor substantially. But our best wishes are for its long life and success.

*The Prescription*, a monthly journal devoted entirely to practical therapeutics, edited by William C. Wile, A. M., M. D., Danbury, Conn. This is a neatly gotten up little journal, and it is certainly practical, and should meet with favor every where. Success to the enterprise.

**Of Interest to Local Medical Societies.**—Not every practitioner is able to purchase a fine microscope; yet the time has come when every one must be able to make microscopic examinations, and the one who is behind in microscopy "is going to get left." It is becoming a popular expedient—as the profession is now pretty well organized and is organizing—to own such things in common—the property of the local society—and every society in Texas should own a microscope, and use it.

The labors of the JOURNAL in behalf of organization, and its defense of rational medicine, are well known. We have done all any man on earth could do for what *some* conceived to be the best interests of the State Association, and now we want the profession to express their appreciation of it by doing something for the JOURNAL'S interest. We want your support in our laudable work, and we ask you to subscribe. As an inducement to work for us, we offer (see advertisement) to donate, as a gift, one of Aloe & Co.'s bran new "Diagnostician" Microscopes, $60, and complete outfit, including a fine Abbe condenser, $15, to the society which, by November 1, will send in the largest list of paid subscribers at $2 each. (This is with the understanding that societies will interest themselves, and compete for it.)

As further inducement to the individual doctor to work, we will give as a premium for the largest list of paid subscribers obtained by one man by November 1, one of Feick Bros'. antiseptic pocket cases, a perfect little gem; price, $11.

Now, gentlemen of the local societies, interest yourselves in the good cause; send in the subscriptions, and let's see which shall be the banner society of Texas, and get these costly presents.

---

**The Medical Department of the University of Texas.**—The JOURNAL has the authority of the honorable President of the Board of Regents—Dr. T. D. Wooten—for saying that with the $30,000.00, appropriated by the last legislature for that purpose, the Medical College (the Medical Department Univ. of Texas) will rapidly be completed, and money enough is available to completely furnish and equip it in a thoroughly first class manner. A Faculty will be chosen this summer, and every man will be eminent and able in his special line. It is to be a high grade school in every particular, and we are authorized to announce that it will be ready for students this fall. Due notice will be given of the opening of the first session, and the Faculty will be announced as soon as selected. There is not, and never has been, any connection between the Texas Medical College and the Medical Department of the University; and our understanding is that with the beginning of the latter the former ceases to be, and will turn over to its successor all buildings, property, etc., now being used by the present college.

With her four hundred students annually it has already ceased to be a reproach to Texas that they must go abroad for a medical education. The good work already done by the Texas Medical College and Hospital will be carried on to splendid results by its successor, and the day is not far distant when the possession of a Texas diploma will be a coveted honor, and a passport to the highest circles of the medical world.

### Notes of the Recent National Medical Convention.

The election of Dr. H. O. Marcy as President of the American Medical Association will be hailed with delight in every section throughout the United States. It is an honor to the Association, for, in every respect, he exemplifies the highest type of the American physician. Texas shakes hands with Massachusetts, and congratulates her on this honor *to the State*.

The Vice-President is the genial and witty Willis P. King, another happy selection, and a compliment to the *South*.

So, the *Journal of the American Medical Association stays*, notwithstanding the tremendous efforts to pull it up by the roots and transplant it at Washington. The trustees had recommended it, but a consensus of the views of its 5400 subscribers was taken—expressed through its columns—and the verdict is Chicago; good enough for the World's Fair; the home of Father Davis, it is good enough for the *Journal*.

The trustees reported in favor of the *Journal* remaining at Chicago, in deference to the wishes of the profession, and advised hat a building for its permanent home be erected as soon as there is money enough in the treasury. The President of the Association recommended that an able editor be employed at a salary of from $10,000 to $15,000 per year.

There was a big fight over the resolution to meet in Detroit, but when Father Davis threw his weight in the scales, down they went.

Dr. J. S. Cain, of Nashville, a courteous, true-born southern gentleman, was chosen to deliver the address in medicine next session. Dr. Cain attended our Fort Worth meeting, it will be remembered, and made many friends.

The time of meeting of the American Medical Association was changed from the first Tuesday in May to the first Tuesday in June. A good move.

A motion to recognize as branches of the A. M. A., all State and Geographic District Medical Associations having a membership of one hundred or more, now in affiliation with the A. M. A., and give members of said Societies all the rights and privileges accorded delegates to the A. M. A., was referred to a committee of five to confer with Societies concerned, as to the desirability and practicability of the measure proposed. We would be glad to see it adopled.

The A. M. A. made a great mistake when they refused to ratify Hot Springs, Ark., as the next place of meeting, and in selecting Detroit, Mich., instead. It is due the South that the Association should meet in striking distance of the extreme Southern States, once in a while. It met in Detroit once before and had a "stormy time"; New Orleans, in 1885. By all means select Galveston next year.

Dr. Frank Woodbury was elected President; Dr. C. H. Hughes, Vice-President; and Dr. Culbertson, of the *Lancet and Clinic*, Secretary of the Association of American Medical Editors.

The South was justly remembered in making Dr. Davis, of Alabama, one of the Vice-Presidents of the A. M. A.

---

"I Find Ponca Compound to act very nicely in inflammation of the cervix uteri and irritation of the ovaries."

E. BRINKERHOFF, M. D.,
Bristolville, Ohio

